# HSP70 mediates a crosstalk between the estrogen and the heat shock response pathways

**DOI:** 10.1016/j.jbc.2023.102872

**Published:** 2023-01-05

**Authors:** Maruhen Amir Datsch Silveira, Fatemeh Khadangi, Sofiane Yacine Mersaoui, Divya Naik, Jean-Yves Masson, Steve Bilodeau

**Affiliations:** 1 Centre de recherche du CHU de Québec – Université Laval, axe Oncologie, Québec, Québec, Canada; 2 Centre de Recherche sur le Cancer de l’Université Laval, Québec, Québec, Canada; 3 Département de biologie moléculaire, biochimie médicale et pathologie, Faculté de Médecine, Université Laval, Québec, Québec, Canada; 4 Centre de recherche en données massives de l’Université Laval, Québec, Québec, Canada

**Keywords:** chaperones, steroids, transcription, feedback mechanism, HSP70 inhibitor, ERα, estrogen receptor alpha, HSF1, heat shock factor 1, HSP70, heat shock protein 70, HSP90, heat shock protein 90, HSR, heat shock response, qPCR, quantitative PCR

## Abstract

Cells respond to multiple signals from the environment simultaneously, which often creates crosstalk between pathways affecting the capacity to adapt to the changing environment. Chaperones are an important component in the cellular integration of multiple responses to environmental signals, often implicated in negative feedback and inactivation mechanisms. These mechanisms include the stabilization of steroid hormone nuclear receptors in the cytoplasm in the absence of their ligand. Here, we show using immunofluorescence, chromatin immunoprecipitation, and nascent transcripts production that the heat shock protein 70 (HSP70) chaperone plays a central role in a new crosstalk mechanism between the steroid and heat shock response pathways. HSP70-dependent feedback mechanisms are required to inactivate the heat shock factor 1 (HSF1) after activation. Interestingly, a steroid stimulation leads to faster accumulation of HSF1 in inactive foci following heat shock. Our results further show that in the presence of estrogen, HSP70 accumulates at HSF1-regulated noncoding regions, leading to deactivation of HSF1 and the abrogation of the heat shock transcriptional response. Using an HSP70 inhibitor, we demonstrate that the crosstalk between both pathways is dependent on the chaperone activity. These results suggest that HSP70 availability is a key determinant in the transcriptional integration of multiple external signals. Overall, these results offer a better understanding of the crosstalk between the heat shock and steroid responses, which are salient in neurodegenerative disorders and cancers.

The heat shock response (HSR) is highly conserved among eukaryotes and triggered by a range of environmental changes to survive harsh conditions (1, 2). In mammals, the master transcription factor orchestrating the HSR is heat shock factor 1 (HSF1), which increases expression of molecular chaperones and other key genes involved in cell proliferation, survival, and energy metabolism (3, 4). Proper regulation of the HSR is essential to regulate protein homeostasis and maintain cellular fitness and survival (1, 5). In addition, alterations of the HSR pathway are associated with aging, neurodegenerative diseases, and cancer (3, 6, 7). Interestingly, multiple pathways, including the steroid and inflammatory signaling, have been shown to promote or interfere with the HSR in a wide range of diseases (4, 5, 7, 8). However, the molecular mechanisms are not always well established.

The HSR relies on chaperones to fine-tune the cellular response to stresses. Indeed, chaperones are required to block the transcriptional activity of HSF1 but are found in a limited cellular pool (9, 10, 11, 12, 13). Stresses increase misfolded proteins and, accordingly, the cellular need for chaperones, freeing up HSF1 to activate transcription (14, 15). This mechanism, known as chaperone titration, is central to the control of the HSR (2, 14). When activated, the HSR increases the pool of chaperones to account for misfolded proteins, but at the same time, creating a negative feedback loop inactivating HSF1 (12, 14, 16, 17). Central to this feedback loop is the availability of the heat shock protein 70 (HSP70), a major component of the network of chaperones (12, 13, 18). HSP70 negatively regulates HSF1 binding to DNA, accelerates HSF1 deactivation, and, accordingly, attenuates the HSR (12, 13, 19, 20). Therefore, the availability of HSP70 is considered a key determinant of the amplitude and duration of the response to stresses.

In absence of stresses, chaperones are playing important roles in the folding/unfolding of proteins and the assembly/disassembly of macromolecules in addition to many other cellular processes (15, 21). Among them, the heat shock protein 90 (HSP90)/HSP70 chaperone machinery is essential for proper response to steroids (21, 22). Indeed, these chaperones are associated with the steroid hormone nuclear receptors, including the estrogen receptor alpha (ERα), progesterone receptor, androgen receptor, and the glucocorticoid receptor. They are important members of the chaperone assembly cycle associated with ligand-free nuclear receptors to maintain proper folding of the ligand-binding domain (21, 23, 24, 25, 26). Upon binding of their steroid ligand, these nuclear receptors translocate to the nucleus to modulate the transcriptional program (27, 28). HSP70 and the other chaperones interact with the nuclear receptors to maintain a primed complex in the cytoplasm (29). Therefore, the presence of a steroid ligand decreases the concentration of cytoplasmic nuclear receptors, breaking the chaperone assembly cycle and leading to increased availability of HSP70 and other chaperones in the cytoplasm (21, 27, 30). Whether the increase in HSP70 availability following hormonal stimulation affects the response to environmental perturbations remains to be determined.

Here, we show that HSP70 mediates a crosstalk between the HSR and estrogen responses. During concomitant HSR and steroid hormone activation, HSP70 is recruited to HSF1 target genes to disengage transcription. We are proposing a model where cellular availability of HSP70 following hormonal stimulation is increasing the negative feedback loop to inactivate HSF1. These results suggest that hormone levels influence the stress response, which could have broad implications for hormonal therapies in cancer and neurodegenerative diseases.

## Results

### Concomitant HS and stimulation of nuclear receptors lead to more HSF1 foci

**Figure 1:**
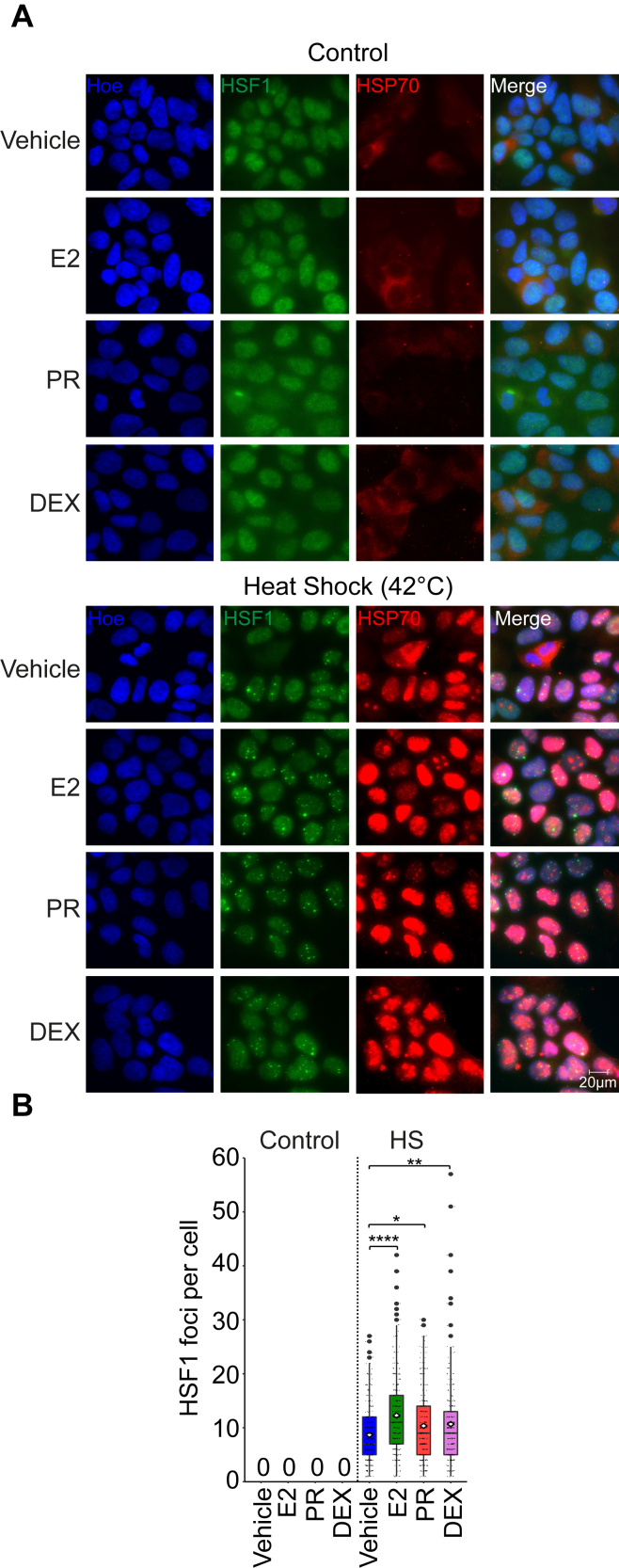
**Stimulation of steroid nuclear receptors by their ligands increases HSF1 foci formation under stress.***A*, representative images from three biological replicates of HSF1 (*green*) and HSP70 (*red*) in MCF7 cells treated with 100 nM of estradiol (E2), 100 nM of progesterone receptor (PR), or 100 nM of dexamethasone (DEX) alone or in combination with a 42 °C heat shock (HS) for 60 min. Hoechst 33342 (Hoe, *blue*) is used to stain the nuclei. Images were taken with a 60× objective, and the scale bar (20 μm) applies to all panels. *B*, box and whisker plots showing the number of HSF1 foci per cell for each condition presented in (*A*) from three independent experiments (N = 100 cells per experiment). The median (*black lines*), the average (*lozenge symbol*), and the standard deviation (*error bars*) are represented. Foci are not observed in the control condition (number = 0). *p* Values were calculated using one-way ANOVA with Dunnett’s multiple comparisons test for ∗*p* < 0.05, ∗∗*p* < 0.01, and ∗∗∗∗*p* < 0.0001. HSF1, heat shock factor 1; HSP70, heat shock protein 70.

To determine the impact of a steroid stimulation on the HSR, we selected MCF7 cells, a breast adenocarcinoma model, as they are responding to multiple steroid signals including estrogen, progesterone, and glucocorticoids (31, 32). The formation of HSF1 foci during the stress response is inversely correlated with HSF1 transcriptional activity, whereas dissolution of the same foci increases HSF1 activity (33). Therefore, to measure the response of the HSR pathway, we quantified the formation of HSF1 foci by immunofluorescence. MCF7 cells stimulated with heat shock showed nuclear accumulation of HSF1 in foci by immunofluorescence (Fig. 1*A*). These foci, which were not observed in control or hormone-stimulated cells, reached an average of 8.68 ± 5.55 per cell after heat shock (Fig. 1*B*). The cell-to-cell variability in HSF1 foci is in accordance with the continuum of response amplitudes observed at the single-cell level (34). Interestingly, the average number of HSF1 foci increased significantly when cells were costimulated by hormones and heat shock compared with heat shock alone (8.68 compared with 12.27 with estrogen, 10.34 with progesterone, and 10.68 with dexamethasone; *p* < 0.05 using a one-way ANOVA followed by Dunnett’s multiple comparisons test) (Fig. 1*B*). HSF1 protein levels were maintained or slightly decreased when MCF7 cells were costimulated with heat shock and hormones (Fig. S1*A*) in accordance with degradation of excess HSF1 in the attenuation phase of the HSR (35). To extend the observation to another environmental stress, MCF7 cells were grown under hypoxia (1% oxygen for 6 h) with and without estrogen before HSF1 foci quantification (Fig. S2). Similar results were obtained: concomitant hypoxia with an estrogen stimulation led to an increase in HSF1 foci compared with hypoxia alone (16.22 compared with 41.11; *p* < 0.001 using a paired two-tailed Student's *t* test). These results establish that under stress response, hormonal stimulations increase the formation of HSF1 foci suggesting faster deactivation of the heat shock transcriptional response.

The feedback mechanism inactivating HSF1 following heat shock involves the chaperone HSP70 (12, 13, 19, 20). Indeed, HSP70 controls the HSR by directly binding to HSF1, repressing the transcriptional activity, and favoring dissociation from DNA targets (12, 19). Since the HSP70 chaperone is also an interactor of inactive steroid nuclear receptors in the cytoplasm (21, 22), we wondered if the hormonal stimulation was sufficient to increase its presence in the nucleus. As expected, the heat shock stimulation led to translocation of the chaperone HSP70 into the nucleus but not the estrogen stimulation (Fig. 1*A*). However, we observed that the concomitant stimulation between heat shock and estrogen, progesterone, and dexamethasone systematically led to a modest increase in the nuclear intensity of HSP70 compared with heat shock alone (Fig. 1*A*). Total HSP70 protein levels were not altered during the experiment (Fig. S1*A*). Therefore, these results establish that during the concomitant heat shock and steroid responses, HSP70 is in the nucleus when HSF1 foci are observed.

### Recruitment of HSP70 is increased at HSF1-regulated genes during a heat shock/estrogen costimulation

**Figure 2:**
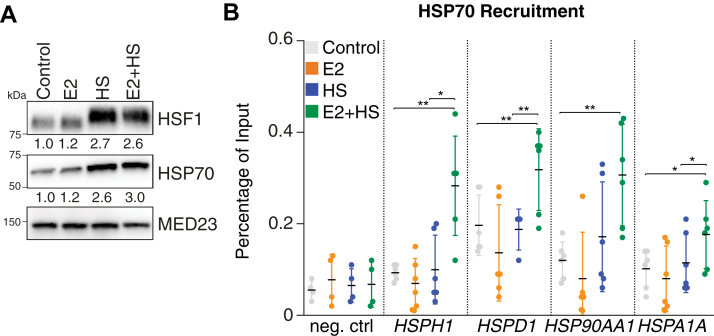
**Concomitant estrogen and heat shock (HS) stimulations lead to increased recruitment of HSP70 to HSF1-regulated genes.***A*, Western blot analysis to measure levels of HSF1 and HSP70 at the chromatin in MCF7 cells treated with 100 nM of estradiol (E2) or a 42 °C HS or both for 60 min. Levels of HSF1 and HSP70 were significantly increased compared with control levels in conditions with HS (*p* < 0.05, biological quadruplicates, paired two-tailed Student's *t* tests). The nuclear protein MED23 was used as a loading control. Protein quantifications were performed using the ImageJ software. *B*, recruitment of HSP70 is increased at HSF1-regulated genes following concomitant stimulations with E2 and HS. The percentage of input immunoprecipitated was measured by ChIP–qPCR in three independent experiments using the promoters of *HSPH1*, *HSPD1*, *HSP90AA1*, and *HSPA1A*, which were all described to be occupied and regulated by HSF1 (37, 38). The mean (*black bars*) and the standard deviation (*error bars*) are represented. *p* Values were calculated using paired two-tailed Student's *t* tests. ∗*p* < 0.05 and ∗∗*p* < 0.01. ChIP, chromatin immunoprecipitation; HSF1, heat shock factor 1; HSP70, heat shock protein 70; qPCR, quantitative PCR.

Since an increase in HSF1 foci was observed in the presence of heat shock and steroids, we hypothesized that the nuclear HSP70 was recruited by HSF1 to the chromatin to deactivate transcription. Interestingly, the biggest increase in HSF1 foci formation during the HSR was observed with the estrogen stimulation, which binds the ERα. To corroborate the immunofluorescence observation and demonstrate HSP70 accumulation at the chromatin, MCF7 cells were stimulated with estrogen, heat shock, and both combined, followed by isolating the chromatin. As expected, the heat shock stimulation increased levels of HSF1 on chromatin (Fig. 2*A*). Levels of HSP70 on chromatin mirrored HSF1 with a reproducible increase observed after heat shock with or without estrogen stimulation. These results support that HSP70 is recruited to chromatin during HSR.

To be involved in the deactivation of HSF1, HSP70 needs to be recruited by HSF1 to regulatory elements. We hypothesized that the increased presence of HSP70 on chromatin could be associated with HSF1-regulated noncoding regulatory regions. We measured recruitment of HSP70 to the chromatin using chromatin immunoprecipitation with a specific antibody followed by quantitative PCR (qPCR) at the promoter regions of validated HSF1 target genes (*HSPH1*, *HSPD1*, *HSP90AA1*, and *HSPA1A*) (36, 37). Basal levels of HSP70 in control conditions were similar to background signal and not influenced by the estrogen stimulation (Fig. 2*B*). Strikingly, for all genes tested, the concomitant stimulation with estrogen and heat shock increased recruitment of HSP70 at the promoters of HSF1-regulated genes (increase ranging from 1.6-fold to 3.0-fold) compared with heat shock alone. In general, HSP70 enrichment over the coding regions was minimal arguing for promoter-specific recruitment (Fig. S3). These observations support a model where the modest increase in nuclear HSP70 observed in a concomitant heat shock/estrogen costimulation is associated with HSF1-controlled regulatory regions.

### The heat shock/estrogen costimulation reduces levels of HSF1 and its coregulators at stress response genes

**Figure 3:**
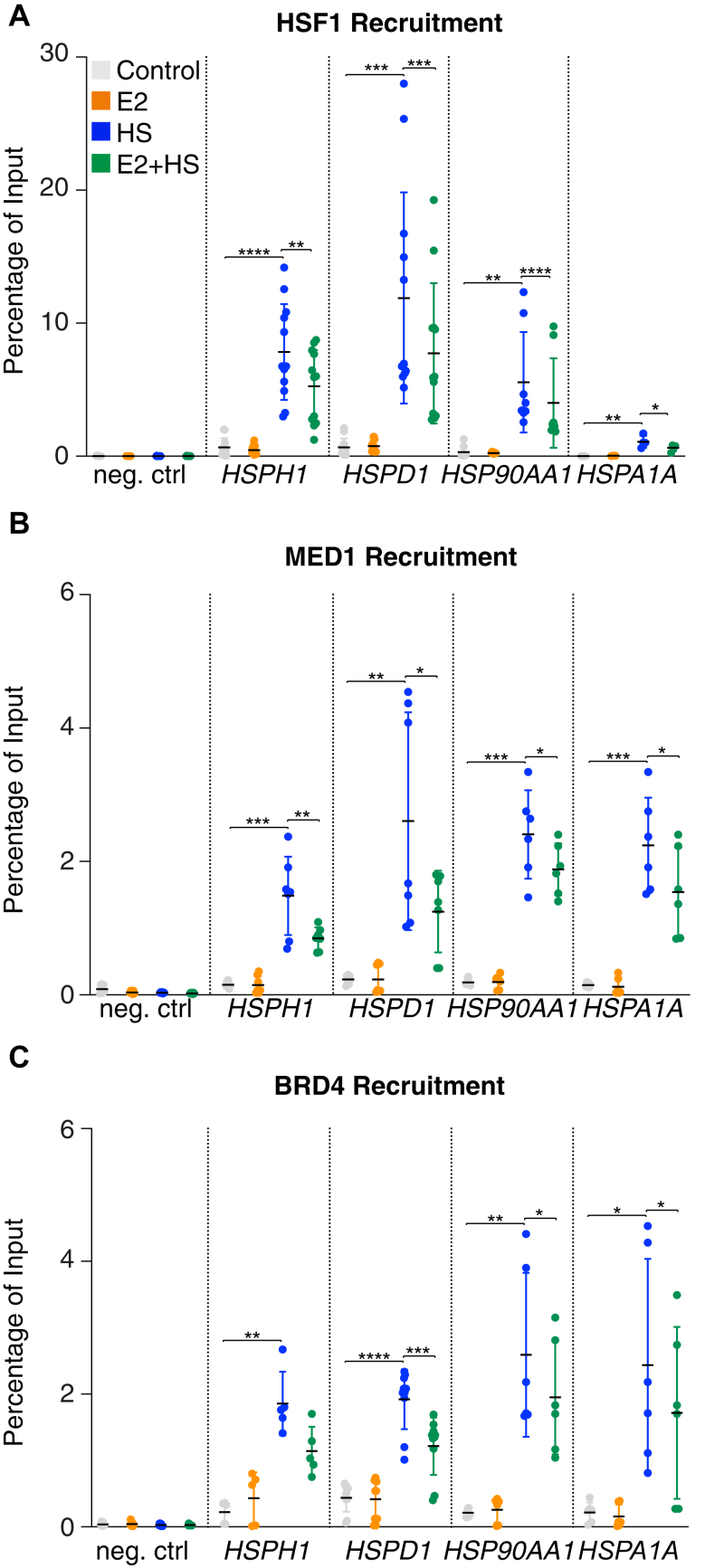
**Recruitment of HSF1 at chromatin is decreased following stimulation with estrogens during stress.** Recruitment of (*A*) HSF1, (*B*) MED1, and (*C*) BRD4 are reduced at HSF1-regulated genes following concomitant stimulations with 100 mM of estradiol (E2) and a 42 °C heat shock (HS) for 60 min. The percentage of input immunoprecipitated was measured by ChIP–qPCR in independent experiments (from 2 to 5) using the promoters of *HSPH1*, *HSPD1*, *HSP90AA1*, and *HSPA1A*, which were all described to be occupied and regulated by HSF1 (37, 38). The mean (*black bars*) and the standard deviation (*error bars*) are represented. *p* Values were calculated using paired two-tailed Student's *t* tests. ∗*p* < 0.05, ∗∗*p* < 0.01, ∗∗∗*p* < 0.001, and ∗∗∗∗*p* < 0.0001. ChIP, chromatin immunoprecipitation; HSF1, heat shock factor 1; qPCR, quantitative PCR.

If the additional recruitment of HSP70 at HSR genes is associated with a feedback mechanism, the occupancy by transcriptional regulators at HSF1-regulated genes will be affected. First, we tested if the heat shock/estrogen costimulation was associated with HSF1 displacement. As shown previously (37, 38), the heat shock stimulation led to recruitment of high levels of HSF1 at the promoter of HSF1-regulated genes (Fig. 3*A*). Interestingly, in all cases, a costimulation with estrogen decreased these HSF1 levels significantly (decrease ranging from 28 to 42%). Total levels of HSF1 were not decreased by the estrogen stimulation compared with heat shock alone (Fig. S1, *A* and *B*) supporting the displacement of HSF1. Previous studies suggested that the estrogen stimulation, through the ERα, leads to phosphorylation and activation of HSF1 in MCF7 cells, and that HSF1 is a transcriptional partner of the ERα (39, 40). These observations imply that the nuclear ERα protein could orchestrate the displacement of HSF1. To test the possibility, we treated MCF7 cells with fulvestrant, a selective ER degrader, and measured HSF1 foci formation. Fulvestrant exposure for 60 min decreased nuclear ERα levels in MCF7 cells (Fig. S4*A*). Degradation of the ERα with fulvestrant, like the estrogen stimulation, increased HSF1 foci when combined with heat shock (10.64 with fulvestrant and heat shock compared with 8.68 for heat shock alone, *p* < 0.05 using a one-way ANOVA followed by Dunnett’s multiple comparisons test) (Fig. S4, *B* and *C*). Similar results were obtained when MCF7 cells were transfected with an siRNA targeting ERα for 3 days with the exception that some HSF1 foci were observed without the heat shock stimulation (Fig. S5). These observations suggest that decreased expression of the ERα triggers similar mechanisms as an estrogen stimulation. Taken together, these results suggest that HSF1 is displaced from the chromatin independently from the nuclear presence of the ERα.

To validate the displacement of HSF1 from the chromatin, we profiled the recruitment of two transcriptional coregulators, MED1 and BRD4, which are known to interact with and function in the HSF1-dependent transcriptional activation (37). Similar results were observed as the heat shock stimulation led to recruitment of MED1 and BRD4 at the regulatory regions of HSF1-regulated genes, whereas the recruitment was decreased when cells were costimulated with estrogen (Fig. 3, *B* and *C*) (decrease ranging from 22 to 52% for MED1 and 25–39% for BRD4). Taken together, these results establish that the additional presence of HSP70 at HSF1-regulated genes is associated with the eviction of transcriptional regulators.

### Inhibition of HSP70 decreases formation of HSF1 foci during concomitant heat shock and estrogen stimulations

**Figure 4:**
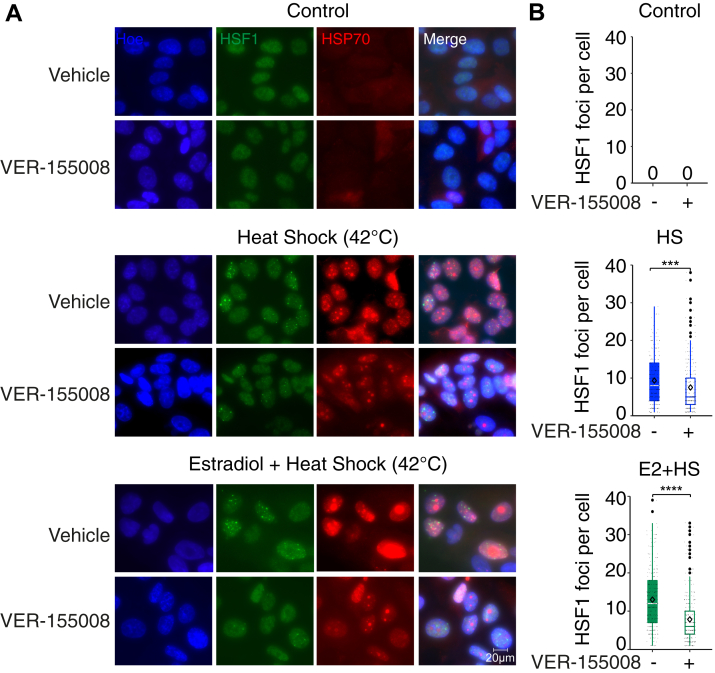
**Inhibition of HSP70 reduces formation of HSF1 foci.***A*, representative confocal images from three biological replicates of HSF1 (*green*) and HSP70 (*red*) in MCF7 cells treated with 42 °C heat shock (HS) alone or in combination with a 100 nM of estradiol (E2) in the presence or the absence of the HSP70 inhibitor VER-155008. Cells were pretreated with the vehicle or the inhibitor (500 nM) for 4 h prior to the HS and E2 stimulations. Hoechst 33342 (Hoe, *blue*) is used to stain the nuclei. Images were taken with a 60× objective, and the scale bar (20 μm) applies to all panels. *B*, box and whisker plots showing the number of HSF1 foci per cell for each condition presented in (*A*) from three independent experiments (N = 100 cells per experiment). The median (*white or colored lines*), the average (*lozenge symbol*), and the standard deviation (*error bars*) are represented. Foci are not observed in the control condition (number = 0). *p* Values were calculated using one-way ANOVA with Tukey’s multiple comparisons test. ∗∗∗*p* < 0.001 and ∗∗∗∗*p* < 0.0001. HSF1, heat shock factor 1; HSP70, heat shock protein 70.

To test the role of HSP70 in the crosstalk between the estrogen and HSR pathways, MCF7 cells were treated with VER-155008 (Sigma–Aldrich Co), a potent and specific inhibitor of HSP70 targeting the nucleotide-binding domain (41), before HSF1 foci quantification. As previously reported (42, 43), treating cells with 500 nM of VER-155008 did not affect HSP70 protein levels (Fig. S1*B*). The increase in HSF1 foci observed following the heat shock, and the heat shock/estrogen costimulation was blocked by the HSP70 inhibitor (Fig. 4, *A* and *B*). Indeed, the number of HSF1 foci observed with heat shock was reduced when cells were pretreated with the HSP70 inhibitor (9.33–7.50, *p* < 0.001 using a paired two-tailed Student's *t* test). As reported in Figure 1, the heat shock/estrogen costimulation led to an increase in HSF1 foci formation (9.33–13.10, *p* < 0.0001 using a one-way ANOVA with Tukey’s multiple comparisons test). This increase in HSF1 foci was abrogated by the VER-155008 inhibitor (13.10–7.83, *p* < 0.0001 using a one-way ANOVA with Tukey’s multiple comparisons test). These results highlight that HSP70-dependent mechanisms are required to increase the number of foci and inactivate HSF1 during concomitant stimulation with estrogen and heat shock.

### HSP70 is responsible for the crosstalk between the estrogen pathway and the stress response

**Figure 5:**
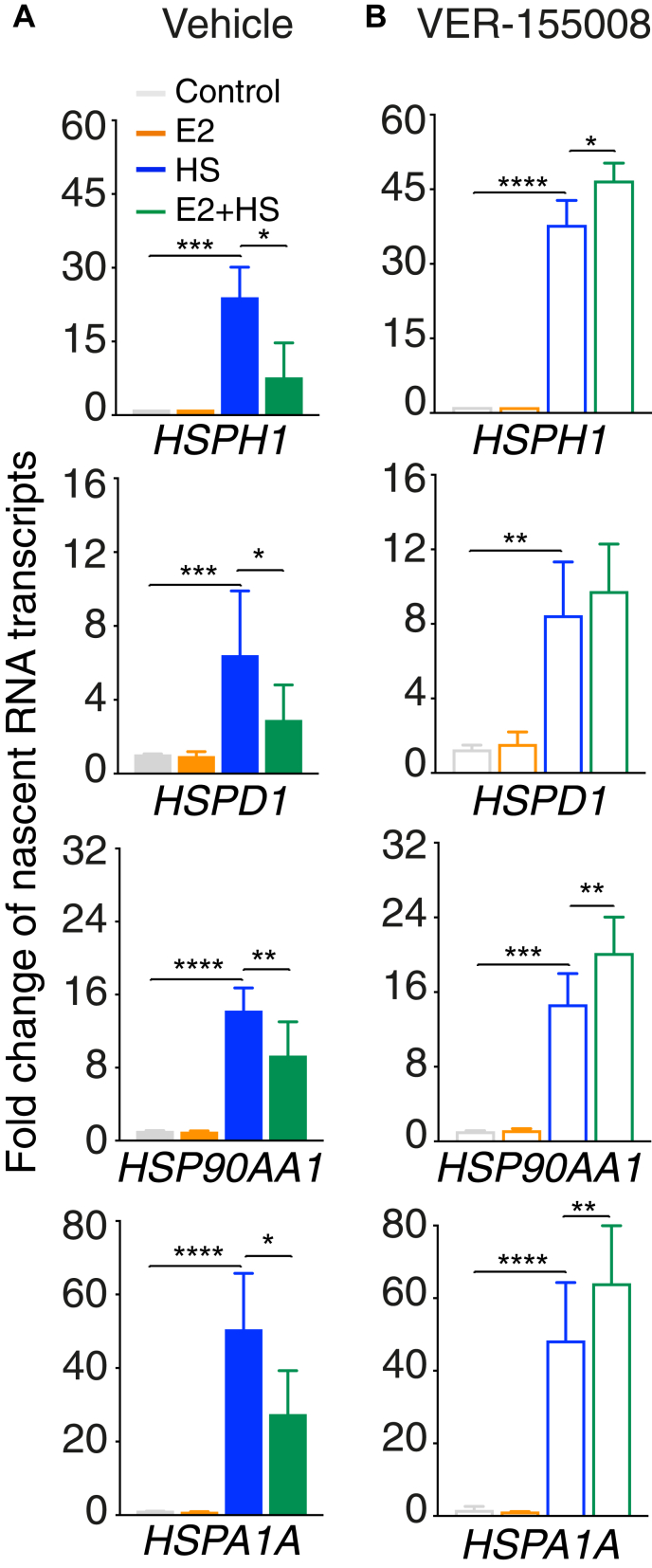
**HSP70 is essential for the transcriptional crosstalk between the estrogen and the stress responses.***A*, stimulation with estradiol (E2) blocks the transcriptional activation of HSR genes. Quantification of nascent RNA transcripts produced in MCF7 cells treated with 100 nM of E2 or a 42 °C heat shock (HS) or both for 60 min. *B*, inhibition of HSP70 reverts the negative effect of the E2 stimulation on the HS transcriptional activation. MCF7 cells were pretreated with 500 mM of the HSP70 inhibitor VER-155008 for 4 h before the E2 and HS stimulations and quantification of nascent RNA transcripts. For (*A* and *B*), nascent mRNA levels were measured in independent experiments (from 3 to 5) using RT–qPCR at HS-regulated genes *HSPH1*, *HSPD1*, *HSP90AA1*, and *HSPA1A*. The mean fold change and the standard deviation are represented. *p* Values were calculated using paired two-tailed Student's *t* tests. ∗*p* < 0.05, ∗∗*p* < 0.01, ∗∗∗*p* < 0.001, and ∗∗∗∗*p* < 0.0001. HSP70, heat shock protein 70; HSR, heat shock response; qPCR, quantitative PCR.

Our results suggest that stimulation of a nuclear receptor modifies the availability of HSP70, exacerbating feedback mechanisms and dampening the stress response. If true, these mechanisms are likely converging on transcriptional control, which is central to the HSR (38). To determine if the estrogen stimulation during the HSR is impacting transcription, we measured the abundance of nascent transcripts produced by HSF1-regulated genes. The heat shock stimulation increased the level of nascent transcripts 6-fold to 50-fold at HSF1-regulated genes *HSPH1*, *HSPD1*, *HSP90AA1*, and *HSPA1A* (Fig. 5*A*). These gains were decreased between 32% and 65% after concomitant estrogen stimulation. To validate that HSP70 was responsible for the crosstalk between the estrogen and the HSR pathways, we next measured nascent transcripts in the presence of the VER-155008 inhibitor. When cells were treated for 4 h with the HSP70 inhibitor before the heat shock, estrogen did not decrease the levels of nascent transcripts produced by HSF1-regulated genes (Fig. 5*B*). Therefore, these results establish that the activity of HSP70 is essential for the crosstalk between the estrogen and HSR, suggesting implication at the transcriptional level.

## Discussion

### HSP70, at the crossroad of multiple pathways

**Figure 6:**
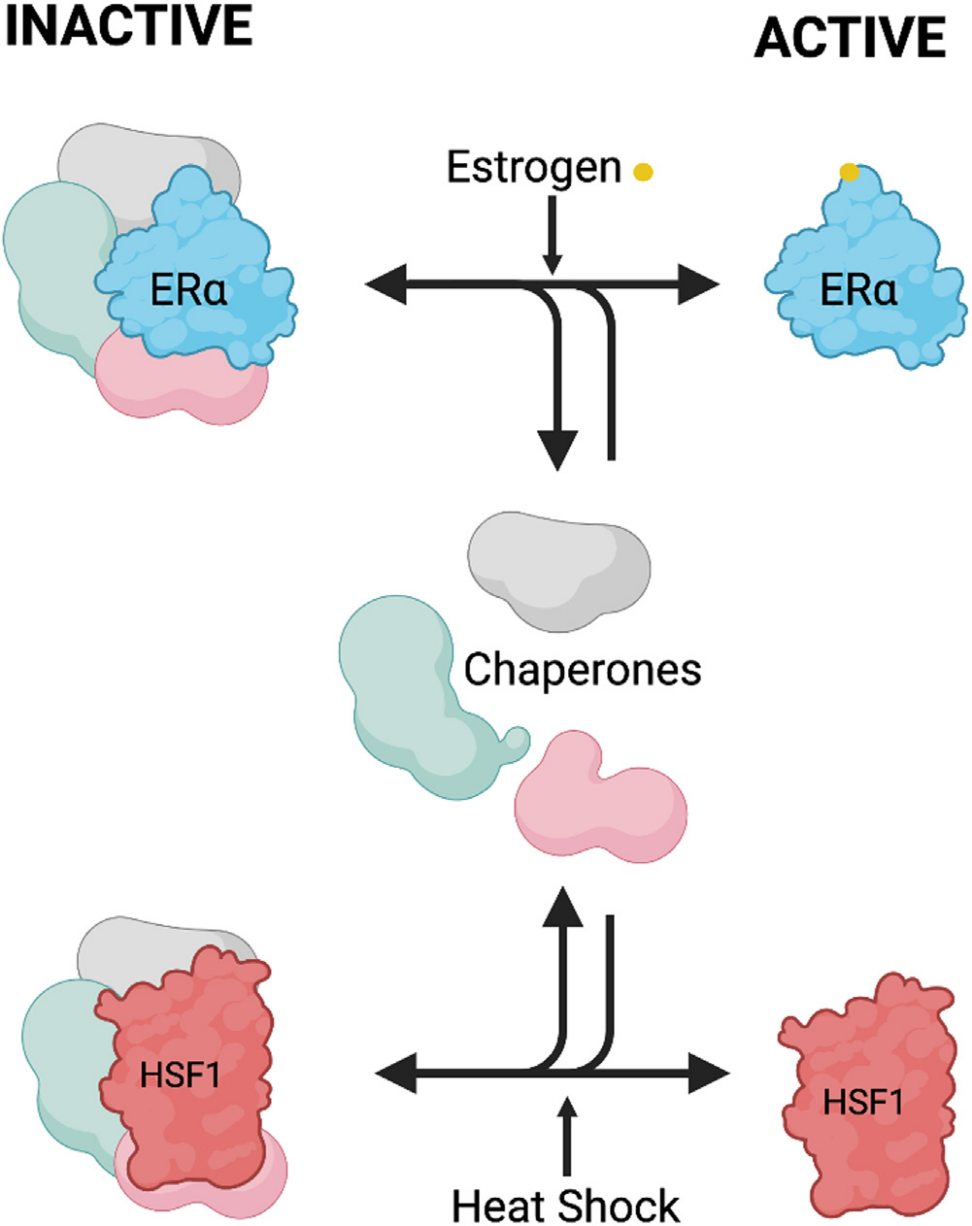
**Availability of HSP70 explains the crosstalk between the estrogen and the stress response pathways.** Chaperones, including HSP70, are sequentially associated with the inactive form of the estrogen receptor alpha (ERα) and HSF1. Upon stimulation with estrogen or heat shock, chaperones not interacting with the transcription factors are creating a pool of available molecules. In the context of a concomitant stimulation of the estrogen and stress response pathways, the pool of free HSP70 is increased leading to amplification of the negative feedback mechanisms on HSF1 and, therefore, a decrease in the transcriptional stress response. HSF1, heat shock factor 1; HSP70, heat shock protein 70.

Availability of chaperones is central to maintain homeostasis in response to external stresses affecting the cell. The concentration of chaperone molecules is constantly regulated at the transcriptional and post-transcriptional levels in various conditions and tissues (15, 44). Our results provide evidence that a steroid stimulation is an additional mechanism to modulate the pool of available HSP70, increasing the negative feedback on the HSR (Fig. 6). When cells are stimulated with steroids, the ligand-bound nuclear receptor translocates to the nucleus, breaking the chaperone assembly cycle in the cytoplasm (21, 27). A decrease in cytoplasmic nuclear receptor increases the pool of HSP70 available to interfere with the HSR. The increased availability of HSP70 is supported by the presence of more HSP70 at HSF1-regulated genes in response to concomitant estrogen and heat shock stimulations (Fig. 2). We are proposing that additional recruitment of HSP70 at the promoter of HSR genes disengages HSF1 leading to decrease of the HSF1-dependent transcriptional program (Figs. 3 and 5). In fact, previous observations support that when the free pool of HSP70 increases, HSP70 binds to the transactivation domain of HSF1 forcing HSF1 from the trimeric (active) to monomeric (inactive) form, releasing it from DNA and leading to proteasome degradation (14, 19). While the stoichiometry of HSF1 *versus* the nuclear receptors remain to be determined, our results support a model where HSP70 availability is an important crosstalk mechanism between the steroid and HSR pathways.

### The cellular capacity to respond to concomitant environmental signals

The capacity of a cell to integrate concomitant environmental signals is important for the physiological and pathological responses. Indeed, the human body is constantly exposed to variable levels of stimuli, responding and adapting with the changing conditions (45, 46). Our model suggests that the ability of a cell to respond to a stress signal depends on the level of the steroid response. For steroids, levels are constantly fluctuating, engaging with their respective nuclear receptors in the cytoplasm. Expression levels of these nuclear receptors are cell type specific (47) and therefore, the proportion of HSP70 engaged with this class of protein will vary from cell to cell. Simultaneously, each cell is expressing various levels of components of the HSR pathway while sensing evolving stresses in real time (44). Therefore, the available pool of HSP70 is constantly fluctuating. If we consider HSP70 as an integrator of concomitant signals balancing the strength of responses, it raises the question whether a steroid response will be favored over the HSR in a physiological context. The answer is potentially complex. Previous reports suggested that steroid stimulations could be attenuating the HSR (48, 49). However, considering that the response will be cell type and context specific, a detailed molecular and biochemical analysis in different conditions will be required to determine the extent of the role of HSP70 as a pathway integrator.

### The importance of the crosstalk between the steroid and HSRs for human diseases

Several neurological disorders are caused by accumulation of misfolded proteins typically associated with the loss of HSF1 function and/or low activity of chaperones (6, 50, 51). For example, pathological accumulation of alpha-synuclein in Parkinson’s disease or the mutated huntingtin protein in Huntington’s disease triggers HSF1 degradation (52, 53). Similarly, the accumulation of protein aggregates titrates chaperones leading to reduced soluble levels and availability (54, 55). Interestingly, overexpression of HSF1 or chaperones often restore normal functions. Indeed, active Hsf1 was shown to suppress the formation of polyglutamine aggregate *in vivo* (56). Furthermore, increased levels of HSF1 reversed the deficiency of Purkinje cells in Alzheimer’s disease (57). Similar observations were made for the chaperone HSP70 as overexpression rescued neurons in Alzheimer and Parkinson disease animal models by blocking or dissolving the aggregation of Tau and alpha-synuclein, respectively (58, 59, 60, 61, 62, 63). Therefore, sufficient available levels of HSR components are required to protect against the accumulation of misfolded proteins and the pathological problems associated.

Our model suggests that under steroid stimulations, cells have an increased availability in chaperones free to interact with other client proteins, potentially misfolded. An extrapolation of our model suggests that diseases associated with low activity of HSP70, and potentially other chaperones could be reverted, partially or totally, by hormonal therapies. Strikingly, epidemiological studies suggest that steroids are playing protective roles in neurodegenerative diseases like Alzheimer’s disease, Parkinson’s disease, Huntington’s disease, and amyotrophic lateral sclerosis (64, 65, 66). These diseases are all associated with low activity of chaperones (6, 50, 51). Neurons stimulated with estrogens were shown to be more susceptible to survive multiple stresses (67, 68). For example, in Alzheimer’s disease, estrogens reduce the amyloid-beta fibrillation *in vivo*, which restores and improves the general cognitive function (69, 70, 71). At the opposite of neurological disorders, in most cancers, chaperone concentrations are increased in response to the numerous mutated proteins allowing tumor cells to survive, grow, and proliferate (7, 72). Therefore, many molecules inhibiting chaperones are being developed for cancer therapy (73). If current hormonal therapies for hormone-dependent cancers are leading to increased chaperone availability, a therapeutic approach including chaperone inhibitors could reveal more effective. Taken together with our results, these observations suggest that hormonal therapies could increase bioavailability of chaperones, modify the physiological response to stresses, and influence disease evolution.

## Conclusions

The discovery of HSP70 as an important protein in the crosstalk between major signaling pathways raises several mechanistic and functional questions. For example, one could imagine that steroid responses have precedence over stress responses. Also, if the bioavailability of chaperones represents an important avenue for the treatment of neurodegenerative diseases, it will be interesting to optimize interventions to maximize their availability. Future experiments will reveal to which extent modulation of the chaperone bioavailability is a potential therapeutic approach.

## Experimental procedures

### Cell culture

MCF7 (American Type Culture Collection; catalog no.: HTB-22) cells were grown in Dulbecco's modified Eagle's medium (Gibco; catalog no.: 11965-092) supplemented with 10% fetal bovine serum (Invitrogen; catalog no.: 12483020), 100 μM minimum essential medium nonessential amino acids (Cellgro; catalog no.: 25-0250), 2 mM l-glutamine (Gibco; catalog no.: 25030-081), 100 U/ml penicillin, and 100 μg/ml streptomycin (Gibco; catalog no.: 15170-063). For hormonal stimulations, cells were kept for 3 days into phenol-red free Dulbecco's modified Eagle's medium (Corning; catalog no.: 17-205-CV) supplemented with 5% charcoal/dextran-stripped fetal bovine serum (Fisher; catalog no.: SH3006803), 100 μM minimum essential medium nonessential amino acids, 2 mM l-glutamine, 100 U/ml penicillin, and 100 μg/ml streptomycin. Cells were treated with β-estradiol (Sigma–Aldrich Co; catalog no.: E8875-250mg), progesterone (Sigma–Aldrich Co; catalog no.: P0130-25g), dexamethasone (Sigma–Aldrich Co; catalog no.: D-1756), fulvestrant (ICI, catalog no.: 182780; ToCris, catalog no.: 1047) or vehicle (ethanol) as indicated. For heat shock, cells were grown at 42 °C for 60 min. For hypoxia, cells were grown at 37 °C in 1% oxygen for 6 h. The HSP70 inhibitor VER-155008 (catalog no.: SML0271) or vehicle (dimethyl sulfoxide) was used as indicated.

### siRNA transfection

MCF7 cells were reverse transfected with the siGENOME Nontargeting siRNA Pool 1 (Horizon; catalog no.: D-001206-13-05) or the siGENOME Human ESR1 siRNA—SMARTpool (Horizon; catalog no.: M-003401-04-0020) using Lipofectamine (Lipofectamine RNAiMAX Transfection Reagent; catalog no.: 13778075) in a 6-well plate according to the manufacturer's protocol. The final siRNA concentration was 50 nM. Fresh media were added after 24 h for an additional 48 h before harvesting.

### Western blot

Cells were harvested in cold PBS and homogenized in radioimmunoprecipitation assay buffer (Biobasic; catalog no.: RB4477) supplemented with protease inhibitors (Roche; catalog no.: 11697498001) for 10 min at 4 °C followed by centrifugation at 12,000*g* for 20 min. For subcellular fractionation, cells were processed as previously described (74). Briefly, cells were lysed with the cytoplasmic lysis buffer (10 mM Tris–HCl [pH 7.9], 0.34 M sucrose, 3 mM CaCl_2_, 2 mM magnesium acetate, 0.1 mM EDTA, 1 mM DTT, and 0.5% NP-40) supplemented with protease inhibitors. Intact nuclei were pelleted by centrifugation at 3500*g* for 15 min. Nuclei were washed with the cytoplasmic lysis buffer without NP-40 and lysed with the nuclear lysis buffer (20 mM Hepes [pH 7.9], 3 mM EDTA, 10% glycerol, 150 mM potassium acetate, 1.5 mM MgCl_2_, 1 mM DTT, 0.1% NP-40, supplemented with protease inhibitors) by homogenization. The nucleoplasm fraction was cleared by centrifugation at 15,000*g* for 30 min, before enrichment of the chromatin pellet in the nuclease incubation buffer (150 mM Hepes [pH 7.9], 1.5 mM MgCl_2_, 150 mM KOAc, 10% glycerol, supplemented with protease inhibitors), with 0.15 unit/l benzonase purity >90% (Novagen; catalog no.: 70746) for 2 h. Samples were cleared by centrifugation at 20,000*g* for 30 min, and the supernatants containing the solubilized native chromatin proteins were collected. Gels were loaded based on protein concentrations or cell numbers for subcellular fractions. Polyvinylidene difluoride membranes were blocked with a 5% solution of nonfat powdered milk or 5% bovine serum albumin proteins in Tris-buffered saline–0.1% Tween for a minimum of 1 h prior to antibody hybridization. The following primary antibodies were incubated overnight at 4 °C: HSF1 (Cell Signaling; catalog no.: 4356, 1:1000 dilution), HSP70 (Enzo; catalog no.: ADI-SPA-810-F, 1:5000 dilution), MED23 (Bethyl Laboratories; catalog no.: A300-425A, 1:1000 dilution), and Vinculin (Sigma–Aldrich Co; catalog no.: V9131, 1:5000 dilution). Anti-rabbit (Jackson Immunoresearch Laboratories, Inc; catalog no.: 111-035-003, 1:25,000 dilution) and antimouse (Jackson Immunoresearch Laboratories, Inc; catalog no.: 115-035-003, 1:25,000 dilution) secondary antibodies coupled to the horseradish peroxidase were used for detection (Clarity Western ECL Blotting Substrates; Bio-Rad, catalog no.: 1705060). Images were captured using a Chemidoc MP Image System (Bio-Rad) and quantified using the Image Lab Software Version 6.1 (Bio-Rad).

### RNA levels

Cells were washed in cold PBS and harvested using the TriPure Isolation Reagent (Sigma–Aldrich Co; catalog no.: 11667157001). RNA samples were purified using the GeneJET RNA Purification Kit (Thermo Fisher Scientific; catalog no.: K0732) and quantified using a NanoDrop 2000 spectrophotometer prior to reverse transcription using the SuperScript VILO Master Mix kit (Thermo Fisher Scientific; catalog no.: 11755500). For nascent transcripts, the Click-iT Nascent RNA Capture Kit (Thermo Fisher Scientific; catalog no.: C10365) was used. Briefly, cells were treated with 0.5 mM 5-ethynyl-2′-deoxyuridine for the indicated times and washed in cold PBS before RNA extraction. After biotinylation, nascent RNA molecules were captured using streptavidin beads and used as a template for complementary DNA synthesis using the SuperScript VILO complementary DNA synthesis kit (Thermo Fisher Scientific; catalog no.: 11754-050). Real-time qPCR was performed using the SYBR Select Master Mix (Thermo Fisher Scientific; catalog no.: 4472920). Primer sequences for each gene are listed in Table S1.

### Chromatin immunoprecipitation

Chromatin immunoprecipitation experiments were performed as described previously (37, 75, 76, 77, 78). Briefly, 50 million cells were crosslinked for 10 min with 1% formaldehyde and quenched with 125 mM glycine for 5 min. Cells were then washed twice with PBS, pelleted by centrifugation at 1350*g* at 4 °C for 5 min, flash frozen, and stored at −80 °C. After cellular lysis, pellets were sheared between 200 and 600 bp using a Bioruptor Sonicator (Diagenode). Chromatin extracts from 15 to 20 million cells were immunoprecipitated overnight at 4 °C with 50 μl of Dynabeads Protein G (Thermo Fisher Scientific; catalog no.: 10004D) saturated with 5 μg of antibodies. The following antibodies were used: HSP70 (Enzo; catalog no.: ADI-SPA-810-F), HSF1 (Cell Signaling; catalog no.: 4356) (36, 37), MED1 (Bethyl Laboratories; catalog no.: A300-793A) (78), and BRD4 (Bethyl Laboratories; catalog no.: A301-985A100) (79). After washes and reverse crosslink, DNA was purified using phenol extraction. Chromatin immunoprecipitation–qPCR were performed using the SYBR Select Master Mix. The primer sequences for each genomic region are listed in Table S1.

### Immunofluorescence

MCF7 cells were seeded on glass coverslips in 6-well plates. After treatment, cells were fixed using 4% formaldehyde for 15 min at room temperature, washed with PBS, and permeabilized with 0.5% Triton X-100 for 15 min at room temperature. Next, the blocking solution (PBS containing 0.1% NP-40 supplemented with 10% goat serum) was added 1 h before the primary antibodies. The following primary antibodies diluted in blocking solution were used overnight: HSF1 (Cell Signaling; catalog no.: 4356, 1:500 dilution). ERα (Millipore; catalog no.: 06-935, 1:1000 dilution) and HSP70 (Enzo; catalog no.: ADI-SPA-810-F, 1:1000 dilution). After washes with PBS containing 0.1% NP-40, the following secondary antibodies were used at room temperature: Alexa Fluor 488 anti-rabbit (Thermo Fisher Scientific; catalog no.: A-11008, 1:500 dilution) and Alexa Fluor 594 antimouse (Thermo Fisher Scientific: catalog no.: A-11005, 1:500 dilution). Nuclei were stained with Hoechst 33342 (Life Technologies; catalog no.: H3570) in PBS containing 0.1% NP-40 (1:15,000 dilution) for 15 min at room temperature. Images were captured using a Leica TCS SP5 microscope with objectives as indicated in the figure legends. Images were captured using the same parameters for all conditions. HSF1 foci were manually counted for 100 cells per replicate.

## Data availability

Data sharing is not applicable to this article, as no datasets were generated or analyzed during the current study.

## Supporting information

This article contains supporting information (two supplemental figures).

## Conflict of interest

The authors declare that they have no conflicts of interest with the contents of this article.

## Supporting information

TableS1
