# Supplementary figures and images for: HSP70 mediates a crosstalk between the estrogen and the heat shock response pathways
Source: J Biol Chem. 2023 Jan 5;299(2):102872. doi: 10.1016/j.jbc.2023.102872 (PMC9926311; doi:10.1016/j.jbc.2023.102872)

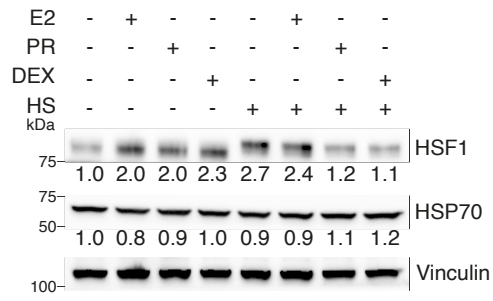**B**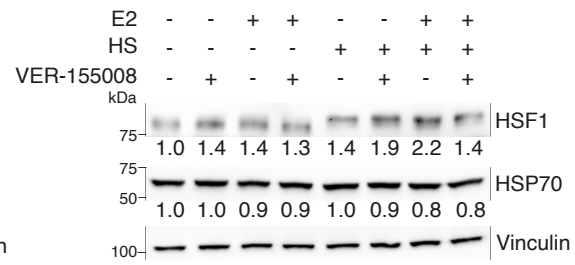

Supplement: Figure S1 [file mmc2.pdf]

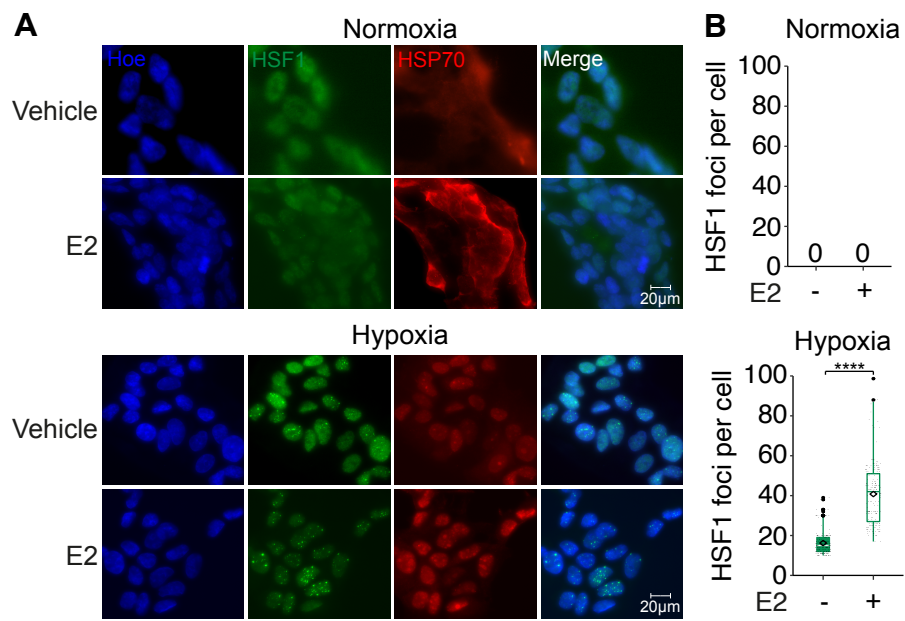

Supplement: Figure S2 [file mmc3.pdf]

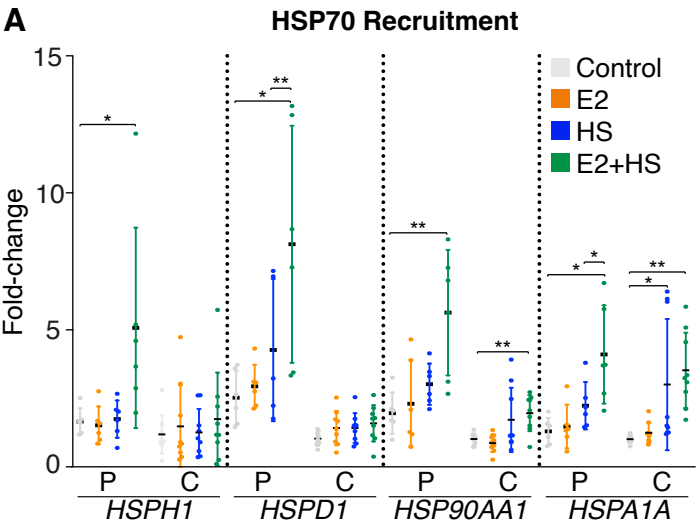

Supplement: Figure S3 [file mmc4.pdf]

**A**

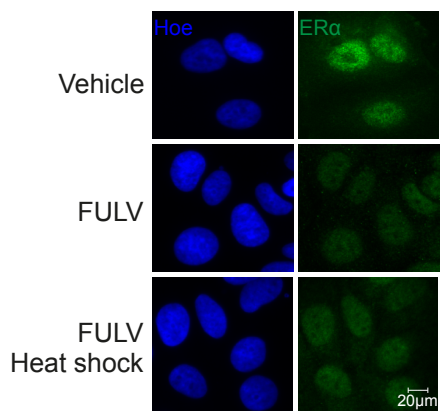

**B**

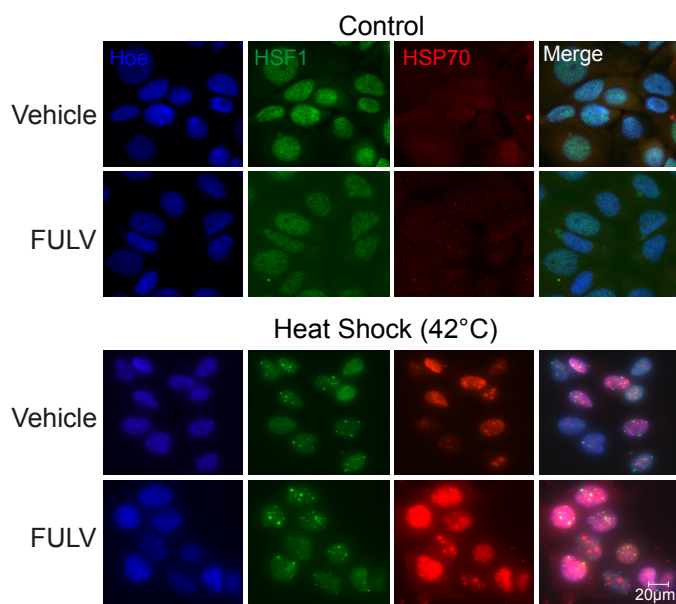

**C**

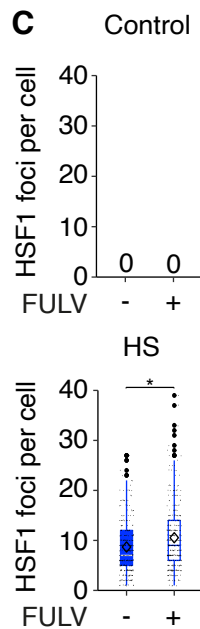

Supplement: Figure S4 [file mmc5.pdf]

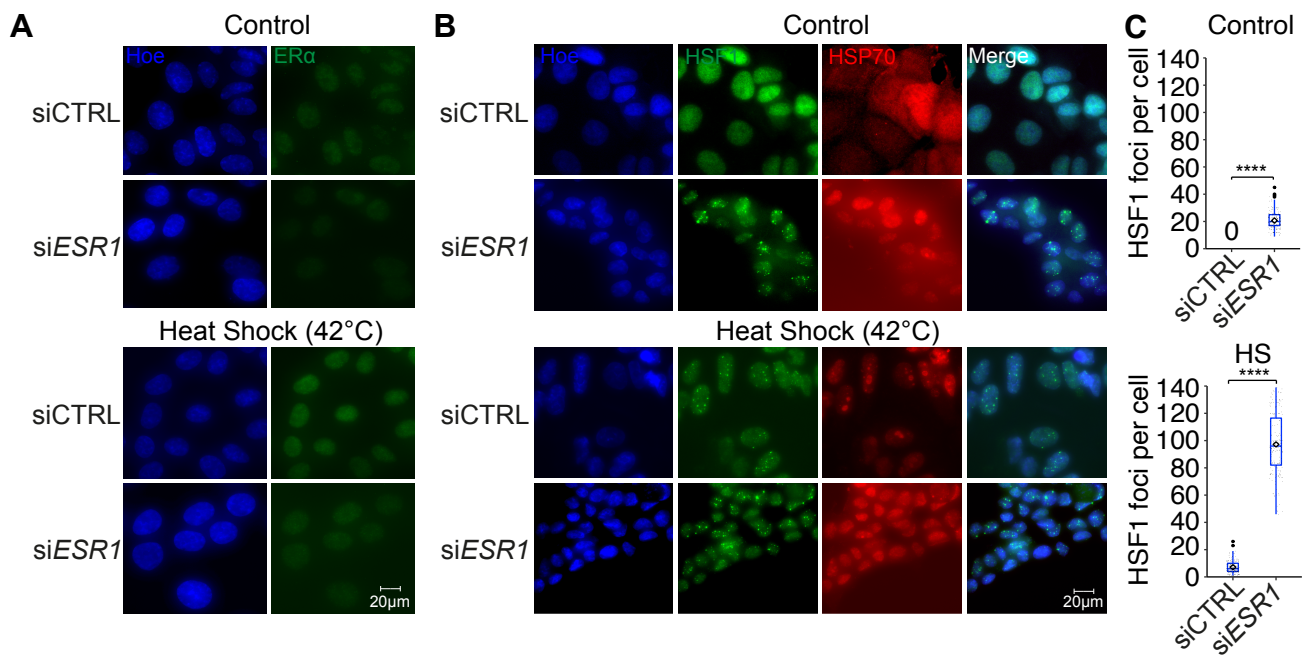

Supplement: Figure S5 [file mmc6.pdf]
